# The Immediate and Delayed Effects of TV: Impacts of Gender and Processed-Food Intake History

**DOI:** 10.3389/fpsyg.2017.01616

**Published:** 2017-09-20

**Authors:** Heather M. Francis, Richard J. Stevenson, Megan J. Oaten, Mehmet K. Mahmut, Martin R. Yeomans

**Affiliations:** ^1^Department of Psychology, Macquarie University, Sydney NSW, Australia; ^2^School of Applied Psychology, Griffith University, Gold Coast QLD, Australia; ^3^School of Psychology, University of Sussex Brighton, United Kingdom

**Keywords:** television, gender, snacking, junk food, habitual diet

## Abstract

Eating while watching TV has generally been found to increase both immediate and delayed energy intake. Here we examine two factors – gender and habitual processed-food intake – that may moderate these effects. Participants [*n* = 153; 95 women, 58 men; *M*_age_ = 19.7 (*SD* = 2.9); *M*_BMI_ = 22.4 (*SD* = 3.1)] ate an *ad libitum* snack either with or without TV, followed around 1 h later by lunch. There was an interaction between TV and gender for both meals. Women tended to consume more snack food in the TV condition, with men consuming more in the no-TV condition. Participants who habitually consumed more processed food also ate more snacks, independent of any other variable, including rated liking. At lunch, men who had earlier snacked with TV ate more than men who had snacked without TV, but this effect was not evident in women. On memory recall, all participants underestimated how much snack food they had eaten, and this was a function of how much they had actually consumed, with greater error only predicted by greater consumption. The results indicate that the effects of TV on eating can vary with gender and that processed-food history can predict snack food intake. While previous findings suggest memory of prior-intake may be impaired by eating while watching TV, the current results suggest this is not necessarily because of TV *per se*, but because people sometimes consume more food under such conditions.

## Introduction

Television (TV) viewing is a significant leisure activity for most Westerners (e.g., Bertrais et al., 2005; Hardy et al., 2006). Many people eat with the TV on and so any effect that TV viewing has on ingestive behavior may have significant impacts on weight gain – and hence obesity – at the population level. Several studies have demonstrated that eating while viewing TV can exert immediate and delayed effects on energy intake (e.g., Bellisle et al., 2004; Blass et al., 2006; Mittal et al., 2010; Ogden et al., 2013; Higgs, 2015). Generally, having the TV on during a meal can increase energy intake relative to a meal eaten alone without TV (e.g., Ogden et al., 2013), although this has not always been observed (e.g., Martin et al., 2009). A further delayed effect of eating with the TV has also been documented. In this case participants consume more energy at a *later* meal, if they earlier ate with TV, which may result from impaired recollection of how much food was eaten with TV (e.g., Higgs and Woodward, 2009; Mittal et al., 2010). In this manuscript we examine two factors that may moderate the impact of TV on immediate and delayed energy intake. The first of these is gender, which as we outline below may affect whether TV alters energy intake or not. The second factor concerns the participant’s habitual consumption of processed food, which may affect their propensity to eat foods commonly consumed while watching TV (i.e., palatable snack foods).

There are two main reasons to consider that the immediate and delayed effects of TV on energy intake may be different for men and women. The first arises from the epidemiological literature that studies the relationship between biological variables (e.g., BMI, blood pressure), gender and hours spent watching TV (Cleland et al., 2008; Parsons et al., 2008; Sugiyama et al., 2008; Wijndaele et al., 2010). It is apparent across several studies that the relationship between time spent watching TV and these biological variables differs by gender: (1) Snack food intake while viewing TV is associated with abdominal obesity in women, but not in men (Cleland et al., 2008); (2) TV viewing in childhood, after controlling for current TV viewing, is predictive of adult BMI in women but not in men (Parsons et al., 2008); and (3) Changes in TV viewing habits (watching more) across time is associated with greater adverse health-related consequences (blood pressure, metabolic syndrome, waist circumference) in women (Wijndaele et al., 2010). Together, these findings suggest that the longer term physiological consequences of TV viewing differ by gender, and that women may be more prone to such consequences than men.

Second, laboratory-based studies examining the impact of TV on food intake are *suggestive* of gender differences. Of the eight studies we could find that compared an eating with TV condition to an eating without TV condition – hereafter the immediate effect of TV – four used women only samples (Bellisle et al., 2004; Ogden et al., 2013; Braude and Stevenson, 2014; Chapman et al., 2014) and four used combined samples of men and women (Blass et al., 2006; Hetherington et al., 2006; Moray et al., 2007; Martin et al., 2009). All four of the women only samples generated the same pattern of outcome with generally more food eaten with TV than without (but see Chapman et al., 2014 – where type of content moderated outcome). This pattern of outcome is different to that of the four remaining studies that used both men and women. Two of these studies failed to find any effect of TV on food intake (Moray et al., 2007; Martin et al., 2009), noting that only Martin et al. (2009) tested for an interaction with Gender – not finding an effect (and reporting no gender difference in cognitive restraint). For the other two, one reported the largest effect size of any TV-related eating study with greater intake in the TV condition (Blass et al., 2006) and the other reported a trend for a greater effect of TV in men, relative to women (Hetherington et al., 2006). This last study also reported no difference in cognitive restraint between men and women. The issue of cognitive restraint is potentially important, as differences on this variable could potentially account for gender-related differences in food intake.

While these findings might lead one to suspect that men and women would respond differently to the immediate effects of TV on energy intake, there is currently no data exploring how they might respond to the delayed effects of TV viewing. Of the three studies exploring the effects of TV on delayed intake, all used women samples (Higgs and Woodward, 2009; Mittal et al., 2010; Higgs, 2015), and found greater food intake in those who had eaten with TV at an earlier meal. Thus, the first aim of the current study was to determine the effects of gender on both the immediate and delayed effects of TV, while taking into account the effects of cognitive restraint and relatedly disinhibition and hunger – all of which may differ by gender (e.g., Carmody et al., 1995; de Castro, 1995; Provencher et al., 2003). These measures were included to ensure that any gender-related effect was not driven simply by gender differences in restraint, hunger or disinhibition.

Our second aim was to explore the effect that a person’s history of processed food intake has on their immediate and delayed response to TV. People have relatively stable dietary patterns, at least over the short to medium term (e.g., 1 year; Feskanich et al., 1993; Crozier et al., 2009) and in around one-third of cases over the longer term as well (e.g., 10 years; Pachucki, 2012). Of particular relevance here are dietary patterns that involve frequent consumption of snack foods, especially those rich in saturated fat and added sugar. Many eating bouts, and especially those involving snack food, are accompanied by TV (Zick and Stevens, 2010). Higher consumption of snack foods is associated with greater TV viewing time (Cleland et al., 2008). Greater TV viewing time is in turn associated with a larger effect of TV on energy intake in the laboratory (Braude and Stevenson, 2014). Moreover, people who report habitually consuming snack foods also tend to eat more of them in experimental settings, either because of greater liking for these foods, a reduced ability to restrain intake, a greater desire to eat them or some combination of these and other factors (e.g., Francis and Stevenson, 2011). For this reason, we also included both processed and unprocessed snack foods for the TV phase of the experiment, as processed snack foods may be especially obesogenic (e.g., via their high palatability). In sum, we predicted that people who habitually consume lots of processed foods might consume more with TV via association (i.e., they may more often snack with TV) – and especially processed snacks – and/or more *in general* (i.e., irrespective of TV), from greater liking, wanting, and less restraint – when confronted with processed palatable snack foods.

A number of studies have suggested that impaired recall of an earlier meal eaten with TV may be responsible for its delayed effect on a later meal (Higgs and Woodward, 2009; Mittal et al., 2010). For this reason, we asked participants at the end of the study to recall what they had eaten during the *ad libitum* snack to see if this was predictive of the amount consumed at lunch. In sum, we suspected that women might be more susceptible than men to the immediate effects of TV, based upon the apparently greater consistency of women-only TV studies. Thus, we predicted greater consumption with TV in women, relative to men. For the delayed effects of TV, while an effect should be present in women, there was no data available to make predictions for men. However, given the hypothesized greater immediate effect of TV in women, this might similarly imply a greater delayed effect in women relative to men. Finally, whether an habitual diet rich in processed foods would be associated with a greater immediate effect of TV (e.g., via association) and/or greater intake in general, has not been tested before.

## Materials and Methods

### Design and Measures

Participants were randomly assigned (using Excel to generate a random sequence by gender) to eat a snack with or without TV. Importantly, this snack phase allowed *ad libitum* consumption, which is the standard approach adopted for immediate TV intake studies (e.g., Blass et al., 2006). This method allowed us to see if either gender or habitual diet influenced the effect of TV on snack food intake. After a delay of approximately 1 h, participants were offered an *ad libitum* lunch, to determine if prior snack intake with or without TV influenced lunch intake. Following lunch participants were asked to recall what they had eaten on the *ad libitum* snack. Consistent with our previous studies (e.g., Mittal et al., 2010), the principal dependent variables were the amount of energy consumed on the snack and lunch meals (noting that the same outcomes obtain if mass eaten is used instead). The between-subject independent variables were gender, processed-food history obtained in the experiment and TV (TV vs. no TV during the *ad libitum* snack).

### Participants

Potential participants were asked to complete the Dietary Fat and Sugar (DFS) questionnaire as part of a broader set of screening measures presented to all 1st-year psychology students. The DFS was used, as it is a validated measure that can reliably discriminate between people who consume higher or lower intakes of saturated fat and/or added sugar – this principally reflecting processed food consumption (Francis and Stevenson, 2013). The pool of potential participants was expanded by advertising on campus, with interested participants completing the DFS in short-form, via the phone. From this pool of potential participants, we identified or estimated (from the short-form) those scoring in the upper and lower quartiles of DFS scores, and they were invited to participate.

One hundred and sixty participants (principally Caucasian [70%] and Asian [25%]) completed the experiment [95 women, 58 men; *M*_age_ = 19.7 (*SD* = 2.9); *M*_BMI_ = 22.4 (SD = 3.1)]. General entry criteria for the study were a history of good health (i.e., no eating disorders, no medications or illnesses likely to affect appetite or cognition), aged 17–30, self-reported normal BMI (noting that in many cases participants estimates were imperfect) and competence in English. Seven cases were not included in the analysis: (1) two participants declined to eat during the *ad libitum* snack; (2) one persistently refused to eat alone in the no-TV condition; (3) two cases were exposed to continuous loud music during testing (an on-campus concert); and (4) two had medical-related histories that precluded inclusion (drug and alcohol use). This left 153 cases for analysis. All participants provided written consent to take part in a study described as studying how diet and eating habits affect behavior. The study protocol was approved by the Macquarie University Human Research Ethics Committee.

### Stimuli

The *ad libitum* snack comprised of six bowls, each of which contained a different weighed and counted portion of food. The foods (and number of units/total energy) were: (1) Pringles chips (20/760 KJ; Pringles Australia); (2) Mars pods (20/1840 KJ; Mars); (3) Cheese bites (20/1380 KJ; Homebrand); (4) Grapes (20/240 KJ; Green seedless table grapes); (5) M&Ms (50/850 KJ; Mars); and (6) Roasted almonds (50/1550 KJ; Homebrand). These snacks were selected so as to present participants with types that would be appealing both to habitual consumers of diets rich in processed foods and to those who consumed processed food far less frequently.

The lunch meal was composed of lasagne [meat (1340 KJ) or vegetarian (1140 KJ) – 260 g portion; Woolworths On The Menu brand], six chocolate Tim-Tam biscuits (1188 KJ; Arnott’s), five chocolate chip cookies (530 KJ; Homebrand) and a sliced apple (176 KJ; Pink Lady), all presented simultaneously.

Participants in the TV group were shown an episode of the light comedy *Friends* (‘The one with all the rugby’; Season 4, Episode 15), which was neither focussed unduly on food nor contained any strong emotive content and was known to appeal an undergraduate demographic (e.g., the youth channel MTV recently started re-showing episodes of *Friends*, significantly boosting their young adult audience).

### Procedure

All participants, tested individually, were instructed to turn off any electronic device and leave these by the entrance to the test area. After participants completed their first *rating set*, composed of evaluations of hunger, fullness, thirst, mood (happy, sad) and arousal (relaxed, alert) on computer presented 100 mm line scales (anchors Not at all to Very) – the snack phase of the experiment began. Participants were seated in a comfortable chair, with the snack food bowls arranged within easy reach, along with *ad libitum* chilled water. All participants were then told: “*Please eat as much of this food as you like. Please ask for more if you want it. All the food that is uneaten will be thrown away*.” No participant requested more snack food. For those assigned to the TV group, the show was started and for those in the no-TV group they were asked to sit quietly for the same length of time that the show ran for (around 22 min). The experimenter then left the room returning at the end of this period. Participants were then asked to complete a second rating set and while they were doing so, the remaining snack food was removed for later weighing.

All participants then engaged in 1 h of neuropsychological testing as part of another study, which served to fill the time between the end of the *ad libitum* snack and the start of the lunch meal. This was followed by the first batch of questionnaires, namely the Depression, Anxiety and Stress questionnaire (DASS; Lovibond and Lovibond, 1995), medical history (including activity levels), and TV viewing habits. Participants then completed their third rating set and this was followed by the lunch meal. As with the *ad libitum* snack, participants were invited to eat lunch, were told that more was available if needed and that all uneaten food would be thrown out (i.e., the same specific instructions as for the *ad libitum* snack were again read out). In this meal, all participants were allowed to read magazines (screened for content) which they did, but no other distractions were present. As with the *ad libitum* snack, *ad libitum* chilled water was provided for drinking. The experimenter left the room while participants were eating, returning after 15 min to check if they had finished. If they had not, they were given a further 5 min, with all participants having completed their lunch meal within this period. This was followed by a fourth set of ratings and while they completed these scales the experimenter removed the remaining food for later weighing.

Participants were then asked to list the food items they had eaten during the *ad libitum* snack (this measure not being used as it had too little variance), which was followed by a cued recall task, in which each snack food name was provided and participants had to indicate how many items of each food they had eaten. Participants were then asked to evaluate how much they had liked the foods presented during the snack and lunch meals [using 100 mm visual analog scales (anchors Strongly dislike, Indifferent, Strongly like)] and about their TV viewing and eating habits (after Braude and Stevenson, 2014). This was followed by the second batch of questionnaires, with all participants completing the DFS (to obtain the most recent information about their consumption of a Western-style diet) and the Three Factor Eating Questionnaire (Stunkard and Messick, 1985), as well as a measure of how much they had liked the TV show in the TV group (liking scale as above). Anthropometric measures were then obtained (height and body weight without shoes), after which participants completed a final set of the rating scales.

### Analysis

All data were suitable for parametric analyses except participants’ age, which required a reciprocal transformation and the snack food memory data, which required a square-root transformation. Data were analyzed using SPSS for Mac version 24.

To determine if there were any differences across experimental groupings in the participant characteristics detailed in **Table 1**, we used a correlational approach. We did so because processed food history was a continuous variable (noting that TV and Gender are bivariate variables), allowing us to use the same approach for all tests. Note that for the bivariate variables, the outcome is identical to an independent *t*-test. To correct for multiple comparisons, alpha was set at 0.007 (i.e., 0.05/7 tests) for each independent variable – TV, Gender and Processed-food history.

**Table 1:** Participant characteristics [mean and (standard deviation)] by experimental grouping (TV vs. no TV) and gender, with range for each variable.

Variable	TV		No TV	
	Women	Men	Women	Men
Number of subjects	48	31	47	27
Age	19.8 (2.7)	21.1 (4.0)	19.0 (2.4)	19.3 (1.8)
Range	17–31	17–32	17–31	17–25
Processed-food intake history (DFS)	60.3 (13.9)	64.1 (14.2)	60.8 (12.5)	63.4 (13.2)
Range	36–99	35–86	38–89	43–89
BMI	22.4 (3.5)	23.0 (3.4)	21.6 (2.6)	23.2 (2.3)
Range	16.0–34.1	16.0–29.1	17.2–30.8	19.6–30.7
Activity	3.7 (2.8)	4.7 (3.1)	3.5 (2.3)	5.7 (3.1)
Range	0–10	0–13	0–8	0–12
TFEQ Restraint	8.1 (4.8)	7.7 (5.0)	7.9 (5.9)	7.3 (5.4)
Range	0–20	1–20	0–20	0–20
TFEQ Disinhibition	7.4 (3.3)	5.6 (2.8)	7.1 (2.7)	6.5 (3.5)
Range	2–15	1–13	1–14	1–13
TFEQ Hunger	7.2 (3.0)	5.6 (2.9)	6.3 (3.4)	7.0 (3.9)
Range	1–13	0–12	0–14	0–14
DASS total	13.7 (10.2)	9.2 (8.1)	10.8 (8.4)	11.5 (8.0)
Range	0–50	0–36	0–42	1–32

As described above, all participants completed the full DFS (i.e., processed food history) either again or for the first time during the experiment. Because this was the most recent measure of habitual processed-food intake this score was used in the analysis. As there was some regression to the mean (for those completing the full questionnaire during recruitment and then later on test) and because half of the participants had only completed the short-form DFS (i.e., those recruited via advertisements), there was a good range of DFS scores. Consequently, DFS score was treated as a continuous independent variable in the analysis. We have used this same approach before (e.g., Attuquayefio et al., 2016; Stevenson et al., 2016) and we note that it is more powerful than grouping, as no information is lost because of aggregation (Preacher et al., 2005).

Intake data were analyzed using ANCOVA, with energy intake at the snack or lunch serving as the dependent variable and Gender, Group (TV vs. no TV during the *ad libitum* snack) and Processed-food history (as a continuous independent variable) as between factors. The covariates used in both ANCOVAs were: (1) BMI as we suspected this would vary considerably within the sample as initial measures were obtained via self-report; (2) The three factors of the TFEQ as these have been identified before as covarying with diet and gender; and (3) Activity levels, as these were found to correlate with Gender (see Results).

As prior studies used a fixed snack/meal with (or without) TV to explore the delayed effects of TV on a later meal – something we could not do because of our interest in both the immediate and delayed effects – we controlled for variation in *ad libitum* snack intake in the analysis (i.e., using it as a covariate). Thus, in the lunch meal analysis, snack food intake was used as an additional covariate.

To examine the effect of type of snack eaten, we calculated the proportion of energy consumed that came from chips and chocolates. This served as the dependent variable for a further ANCOVA using the same design as the snack food intake analysis. In addition, we also examined the impact of snack food choice on lunch intake, this time adding proportion of energy consumed from chips and chocolate on the snack, as a further covariate in the lunch analysis ANCOVA.

Finally, only relevant parts of the rating set data are reported, as overall these provided little additional information beyond the expected pattern of changes. That is all participants decreased in hunger across the experiment [rating sets 1–5, respectively, *M* (*SD*)]; 60.3 (22.3), 34.4 (20.2), 48.0 (20.9), 7.5 (10.4), 13.2 (17.8)] and increased in fullness (rating sets 1–5, respectively, *M* (*SD*); 24.6 (20.6), 56.2 (23.2), 44.8 (21.9), 87.5 (13.8), 82.5 (19.1)].

## Results

### Participants

Participant characteristics by group are displayed in **Table 1**. The TV grouping was found not to correlate with any of the variables in **Table 1**. Gender correlated with activity levels, these being higher in men [*r*(153) = 0.26], with no other significant associations. For Processed-food history, there was a significant negative association with TFEQ Restraint [*r*(153) = -0.30], with higher restraint associated with a diet reportedly lower in processed food. No other associations were significant.

### Immediate Effects of TV on Snack Food Intake

**FIGURE 1:**
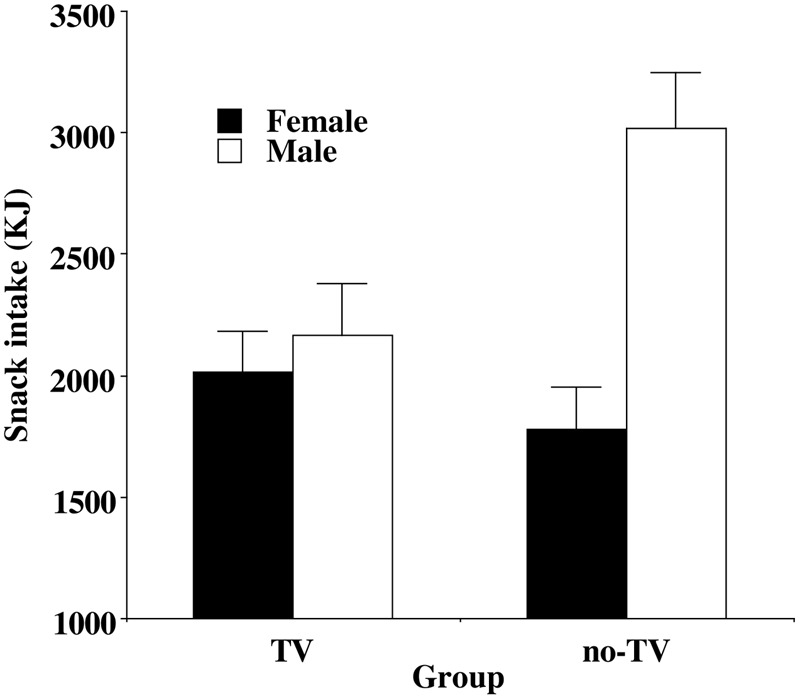
Mean (and standard error) energy intake on the snack meal for women (no significant difference by Group) and men (significantly different by Group).

These data were analyzed using a three-way ANCOVA and two basic findings emerged. The first concerned the TV Grouping and Gender. There was a main effect of Gender [*F*(1,140) = 10.27, *p* < 0.002, $\eta_{p}^{2}$ = 0.07], with men eating more than women. Gender interacted with Group [*F*(1,140) = 7.18, *p* < 0.01, $\eta_{p}^{2}$ = 0.05], which is illustrated in **Figure 1**. We then examined whether the predicted immediate effect of TV was present in women and in men. Although women appeared to consume more of the snack food in the TV group relative to the no-TV group – see **Figure 1** – this difference was not significant (*p* = 0.25). In contrast, men in the TV group consumed significantly *less* snack food than those in the no-TV group (*p* = 0.017).

**FIGURE 2:**
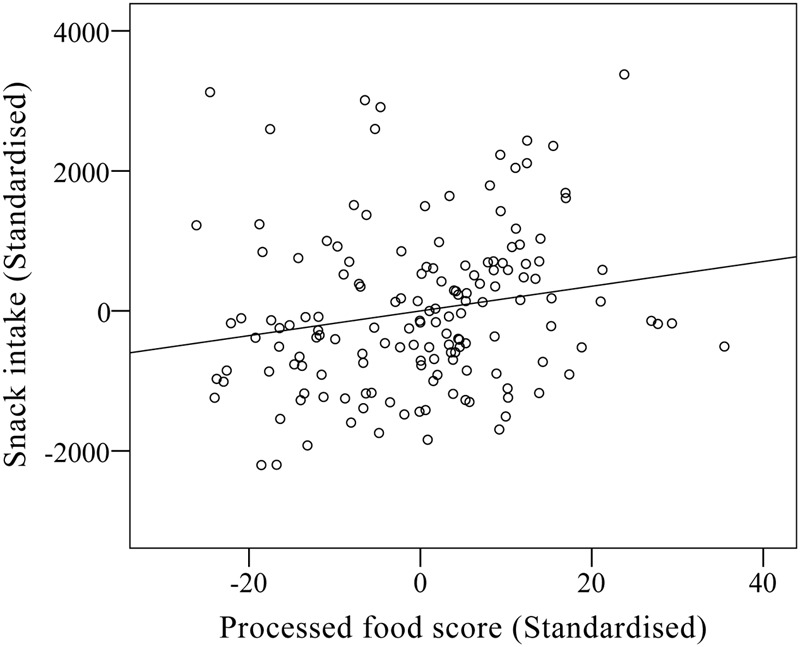
Partial regression plot of processed food score and snack intake.

The second basic finding concerned Processed-food history. There was a main effect of this variable [*F*(1,140) = 5.43, *p* < 0.025, $\eta_{p}^{2}$ = 0.06], which is illustrated in **Figure 2**. Participants with a self-reported history indicative of greater processed food consumption, ate more snack food than participants with a history of lower intake of such foods. Processed-food history did not interact with any other variable.

### Immediate Effects of TV on Type of Snack Food Intake

The proportion of snack food intake that was processed food (i.e., chips and chocolates) – see **Table 2** – was also analyzed using a three-way ANCOVA. This revealed two effects. First, a main effect of Processed-food history [*F*(1,140) = 4.76, *p* < 0.05, $\eta_{p}^{2}$ = 0.03], indicating a greater proportion of processed snack food was consumed by those who also reported eating more processed food habitually. Second, there was a significant interaction between Processed-food history, TV grouping and Gender, [*F*(1,140) = 3.90, *p* < 0.05, $\eta_{p}^{2}$ = 0.03]. To unpack the interaction, we examined these data separately by Gender. For women, there was a non-significant tendency for proportionally greater consumption of processed snack food in the TV group (*p* = 0.089). For men, there was a non-significant tendency for proportion of processed snack food consumption to be moderated by TV grouping, with greater proportional consumption in those who habitually consume processed food and who watched TV, and in those who do not habitually consume processed food and did not watch TV (*p* = 0.065).

**Table 2:** Consumption of processed and non-processed foods [mean (standard deviation)] during the snack phase.

	TV		No TV	
Variable	Women	Men	Women	Men
**Energy (KJ)**				
Processed	1274.6 (858.6)	1165.0 (908.0)	980.2 (763.0)	1468.9 (880.9)
Non-
processed	755.5 (579.8)	1024.8 (749.3)	794.9 (506.2)	1515.1 (925.5)
**Proportion processed (%)**				
	59.0 (24.4)	50.4 (26.5)	49.8 (26.8)	48.7 (21.3)

### Delayed Effects of TV on Lunch Intake

**FIGURE 3:**
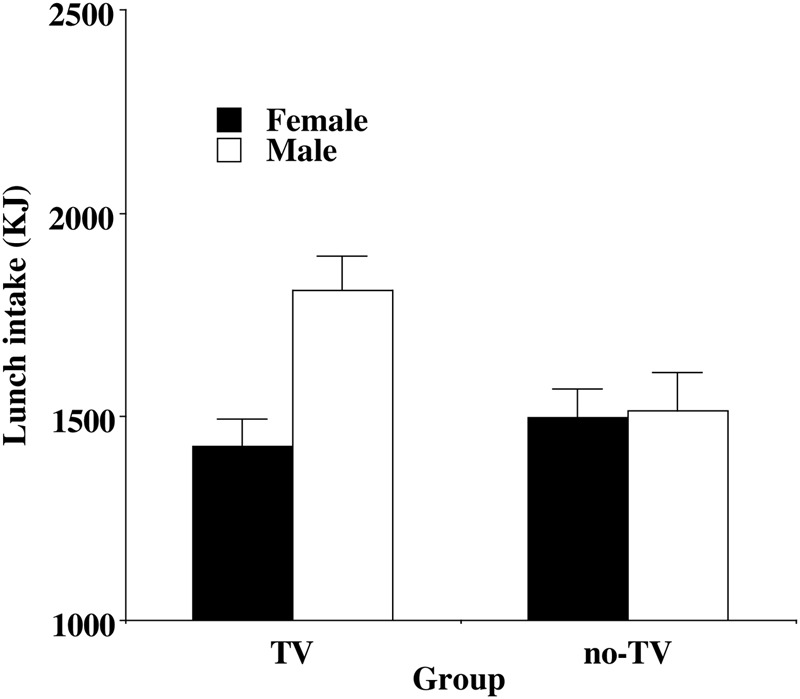
Mean (and standard error) energy intake on the lunch meal for women (no significant difference by Group) and men (significantly different by Group).

The three-way ANCOVA design was also used to analyze the lunch intake data, with one modification namely the addition of the snack food meal intake as a further covariate. The analysis revealed two effects. First, a main effect of Gender [*F*(1,139) = 5.52, *p* < 0.02, $\eta_{p}^{2}$ = 0.04], with men again eating more than women. Second, an interaction of Gender and Group [*F*(1,139) = 4.57, *p* < 0.05, $\eta_{p}^{2}$ = 0.03], which is illustrated in **Figure 3**. Again, we checked to see if the delayed effect of TV was present within each Gender. For women, there was no difference in food intake between the TV and no-TV group (*p* = 0.56), while for men there was significantly greater intake in the TV group relative to the no-TV group (*p* = 0.029). That is men who had snacked with TV ate more at lunch than men who had snacked without TV – even after controlling for earlier snack food intake.

### Effect of Type of Snack Food on Lunch Intake

We repeated the analysis above, now adding in the proportion of snack food intake that was processed food (i.e., chips and chocolate) as a further covariate, but this had no effect on the outcome, with Gender, and Group by Gender, still significant.

### Gender-Related Effects of TV

Next, we examined whether men and women performed differently on other measures that might potentially explain the observed differences in their response to the immediate and delayed effects of TV.

First, we examined whether the nature of the TV show might have influenced performance. While men reported liking the show (*M*_liking_ = 72.7/100), women liked the show more [*M*_liking_ = 83.0/100; *t*(77) = 2.74, *p* = 0.008]. However, show liking did not correlate with snack or lunch intake, either overall (men and women combined), or for either gender alone.

Second, we tested if mood/arousal differences between genders might be relevant, by examining whether these variables differed between men and women across the snack and lunch phase of the experiment. Men and women reported similar changes in mood/arousal states, characterized by increased happiness and relaxation following each eating bout (see **Table 3**).

**Table 3:** Initial motivational state and changes in mood and arousal [mean and (standard deviation] by experimental grouping (TV vs. no TV) and gender.

Variable	TV		No TV	
	Women	Men	Women	Men
**Initial motivational state**				
Hunger	60.8 (24.1)	54.4 (25.2)	62.3 (21.2)	62.6 (16.8)
Fullness	24.1 (17.3)	25.3 (24.0)	25.2 (24.2)	23.6 (15.1)
**Mood and arousal**				
**Snack**				
Before happy	74.9 (15.2)	75.4 (16.6)	74.8 (19.2)	76.3 (13.3)
Before relaxed	63.5 (22.4)	73.4 (22.1)	64.2 (26.1)	66.1 (20.2)
After happy	81.4 (15.4)	81.9 (14.2)	73.9 (17.1)	73.5 (14.9)
After relaxed	77.3 (16.9)	81.5 (16.5)	73.1 (21.7)	72.0 (16.4)
**Lunch**				
Before happy	70.8 (19.8)	70.1 (23.0)	68.9 (21.0)	71.2 (15.3)
Before relaxed	69.8 (20.1)	71.8 (22.1)	65.4 (22.5)	68.7 (19.6)
After happy	79.5 (19.3)	77.9 (19.5)	78.3 (18.0)	76.9 (16.2)
After relaxed	77.9 (16.7)	79.4 (19.7)	75.0 (20.2)	71.7 (22.8)

Third, we looked to see if hunger and fullness ratings might reveal differences in motivation to eat prior to the start of the study (see **Table 3**). There were no differences in hunger or fullness by Gender or Group (or by Group by Gender) at the start of the study, and noting that initial hunger and fullness ratings were not predictive of intake on the snack or at lunch.

Finally, we examined whether TV viewing and eating habits were associated with gender (or group by gender). There were no significant effects. Both men and women reported similar amounts of TV (*M* = 6–10 h per week) and other screen time (*M* = 6–10 h per week) viewing, as with eating with TV (*M* = Once per week) and eating with other screen time (*M* = Once per week).

### Processed-Food Intake History and Snack Consumption

A history of greater self-reported processed-food intake was associated with greater snack intake in the experiment and we examined whether the hedonic explanation briefly identified in the Introduction could account for this finding. First, we determined if this effect applied equally to all of the snack foods. Higher DFS score was positively associated with greater consumption of Pringles [*r*(153) = 0.20, *p* < 0.02], pods [*r*(153) = 0.22, *p* < 0.01], and M and M’s [*r*(153) = 0.26, *p* < 0.001], but not with consumption of almonds (*p* = 0.92), grapes (*p* = 0.65), or cheese (*p* = 0.13). These correlations suggest that it was greater consumption of less healthy snack foods that drove the association between overall energy intake on the *ad libitum* snack and DFS score.

Second, we checked to see if participants self-reported liking for the snack food was predictive of intake. Collapsing across Pringles, pods and M and M’s (given their similar relationship with the DFS score), greater liking for these foods was significantly associated with greater consumption [*r*(153) = 0.17, *p* < 0.05]. We then examined whether DFS score (i.e., frequency of consumption of such foods) was a better predictor of snack intake than participants liking rating. After partialling out liking, the association between Processed-food history and consumption of the less healthy snack foods (collapsing across Pringles, pods and M and M’s) was still significant [*r*(150) = 0.27, *p* < 0.001]. This suggests that greater consumption of the less healthy snacks was better predicted by a history of consuming similar foods before than by how much these snacks were liked.

Finally, we examined whether TV viewing and eating habits were related to Processed-food history, which might be expected based upon previous findings. Higher intakes of processed foods were weakly but non-significantly linked to greater TV viewing time (*r* = 0.15, *p* = 0.06) and eating with TV (*r* = 0.15, *p* = 0.057), and positively but not significantly with other screen time viewing (*r* = 0.13, *p* = 0.12) and eating with other screen time (*r* = 0.07, *p* = 0.37).

### Memory for the *Ad libitum* Snack

Previous findings have suggested that poorer recall of an earlier meal eaten with TV may be associated with greater intake on a later meal (Mittal et al., 2010). Here we examined whether this was also the case and more generally (i.e., *post hoc*) explored participants recall of their snack.

Participants were presented with 180 individual items of food during the *ad libitum* snack (i.e., 20 Pringles, 20 pods, 20 cheese bits and 20 grapes, and 50 M&Ms and 50 roasted almonds). On average each participant consumed 60.8 items (*SD* = 31.0), and recalled consuming (when asked for a number for each food item) an average of 43.5 items (*SD* = 20.9). The difference between actual and recalled consumption was significant [paired samples *t*-test, *t*(152) = 10.79, *p* < 0.001], with participants underestimating their consumption by around 30% on an item basis. It should be noted that while recall accuracy was poorer for M&Ms and almonds (i.e., more of these small items were presented and eaten) the same pattern of outcome is evident for each individual snack food, which is why they are treated together here.

**FIGURE 4:**
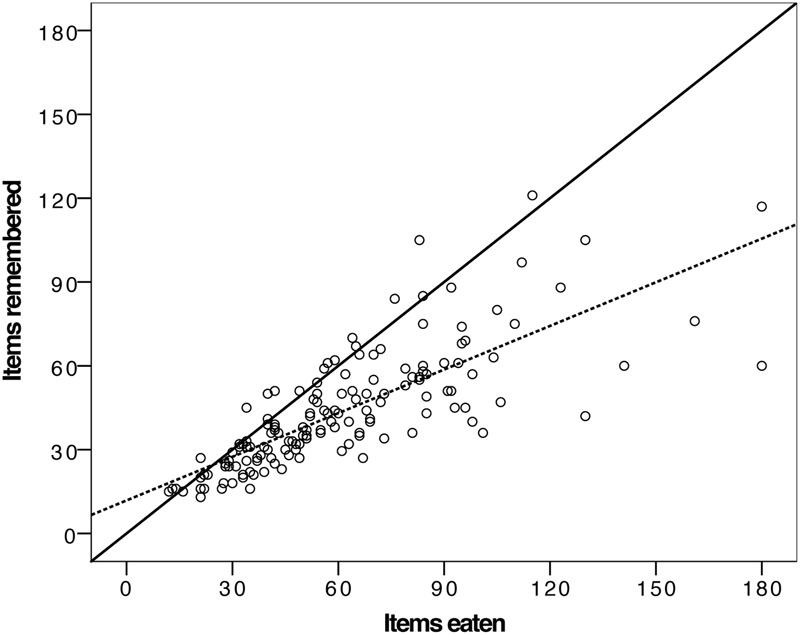
Scatter plot of actual items of snack food consumed against recalled items, with the solid like showing a hypothetical perfect correspondence and the hashed line the actual fitted relationship between these two variables.

We then plotted actual against recalled consumption (see **Figure 4**). Although the underestimation is readily evident (compare the hashed fitted line for these data to the solid *y* = *x* line), it is apparent that the degree of underestimation is a function of the amount consumed, and that greater consumption is predictive of greater under-reporting of intake. To confirm this impression, we calculated the absolute difference between actual and recalled intake and correlated this with actual consumption. This revealed a significant association [*r*(153) = 0.79, *p* < 0.001], indicating that greater consumption of the *ad libitum* snack was associated with greater absolute deviation of remembered from actual intake. That is the more snack food one ate, the greater the degree of recall inaccuracy, with the inaccuracy being underreporting of actual intake.

Finally, we examined whether participants recall of the snack phase was associated with their lunch intake. This was explored using the same ANCOVA design used for the lunch intake data, but with the absolute memory difference score now serving as the dependent variable. The ANCOVA revealed no significant effects of any independent variable. Thus recall performance was similarly inaccurate across all participants irrespective of Gender, TV grouping or Processed-food intake history.

## Discussion

This study examined how gender and processed-food intake history interact with TV viewing to affect energy intake, both immediately, and after a delay. We found that snacking with or without TV had different immediate effects on men and women. There was a significant interaction of TV and gender on snack food intake, even after controlling for individual differences in dietary restraint, disinhibition and hunger. Women tended to consume more food when snacking with TV relative to men, who tended to consume more food when snacking *without* TV. In addition, we also explored the delayed effects of TV on a subsequent lunch meal. We again observed an interaction between gender and the effects of TV. Here, men who had earlier snacked with TV consumed significantly more food at lunch than men who had snacked without TV – as observed before (Higgs and Woodward, 2009; Mittal et al., 2010; Higgs, 2015) – but there was no effect in women. Women consumed the same amount of lunch irrespective of whether they had snacked with or without TV.

For processed-food intake history, the principal finding was that participants who reported a habitual diet richer in processed foods – irrespective of gender – consumed more of the snack foods, than participants reporting diets lower in processed foods. This effect was not better explained by the degree to which participants reported liking the snack food even though liking was related to intake. We also examined the type of snack food participants consumed. Here habitual processed-food intake disposed toward consuming more of the processed snack foods relative to unprocessed snacks. In addition, choice of snack type also interacted with TV grouping, gender and processed-food history. Although this effect was significant, it had a small effect size, and when we examined separately by gender, differences by TV viewing and processed-food history were only marginally significant.

The study also explored the possible origins of the observed gender differences. We could immediately exclude known gender differences in dietary restraint, hunger and disinhibition (e.g., Carmody et al., 1995; de Castro, 1995; Provencher et al., 2003) as we controlled for these variables. There were also no gender-related differences in mood, arousal or initial levels of hunger and fullness. A further candidate was pre-existing screen-time habits, which are known to differ between men and women (Cleland et al., 2008; Parsons et al., 2008; Sugiyama et al., 2008; Wijndaele et al., 2010). Although we did not observe any gender-related effects here, viewing-related habits were weakly – but not significantly – related to participants processed-food intake. Finally, we examined whether participants enjoyment of the TV show might account for gender differences. Although all participants liked it, women liked it more. However, liking the show was not predictive of snack intake.

We suggest two possible causes for gender differences in the immediate effects of TV on snack food intake. The first concerns TV content. A number of groups have shown that content can differentially affect intake. Tal et al. (2014) found that a highly exciting and fast paced movie elevated snack food intake relative to viewing an interview, and there was some indication that this effect was more accentuated in men than in women. Just as different sorts of content – boring vs. engaging – can impact how much people eat (e.g., Mathur and Stevenson, 2015), content might significantly interact with Gender, but there has as yet been no formal test of this idea. A second possibility concerns the nature of the food provided. We used a mixture of processed and non-processed snacks, while a number of previous studies, including our own, have used just processed snacks (e.g., Ogden et al., 2013; Mathur and Stevenson, 2015). Notably, when examining the proportion of processed snack foods consumed, there was a non-significant tendency for women to consume more processed snack food when snacking with TV relative to no TV. This relationship (again a non-significant tendency) was much more complex in men. Here, processed food intake history moderated the effect. These new findings suggest that type of snack food may be an important variable to manipulate in future studies, especially because processed snack foods are highly palatable, energy dense and may often be eaten with TV.

Turning to the delayed effects of TV, it is important to acknowledge that we used a different design to previous studies. Our participants had *ad libitum* access to the snacks. While, we statistically controlled for differences in snack food intake, this earlier *ad libitum* access may have interfered with detecting the delayed effect of TV. If this were the case, then the interference was presumably restricted to women, as men revealed a pattern of outcome consistent with previous findings. Perhaps *ad libitum* access to snack food is more salient in women (relative to men) making them more vigilant about their food intake later in the experiment. It is also important to acknowledge two further methodological issues that might have affected behavior on the lunch meal. First, while unlikely, it is possible that the neuropsychological testing prior to lunch may have had different effects on each gender, thus affecting their respective lunch intake. Second, it is possible that providing participants access to magazines at lunch – distraction – may have differentially affected men and women’s food intake. However, we note that this material is likely to be less distracting than TV and that the gender-related effects here did not resemble those of the snack meal.

Habitual consumption of processed foods was associated with elevated intake of snack food, and especially processed snacks. We explored one potential reason for this, namely greater liking for snack foods in people who report habitual consumption. However, we found that the relationship between snack food intake and processed-food intake history remained significant even after partialling out variance accounted for by liking these foods. Needless to say, it may be that if we had taken more specific measures of liking (i.e., on a food-by-food basis) we might have found evidence that greater liking drives greater intake. However, we note that the processed-food intake measure is based on consumption frequency for a far broader set of processed foods, all characterized by high levels of saturated fat and/or added sugar, and not specifically those used here. So, while we cannot rule out greater liking as an explanation, it is not well supported by the data we have.

A further perspective on processed food history’s impact on snack food intake is also possible. Participants who routinely consume foods rich in saturated fat and/or added sugar may have a pre-existing lower ability to resist them. Several studies suggest that individuals who routinely consume high palatability diets are more impulsive and the weight of evidence suggests that greater impulsiveness probably drives overconsumption of these types of food (see Stevenson, 2017). It is also possible to view these findings from the perspective of incentive salience theory (Robinson and Berridge, 1993). Frequent consumption of highly palatable foods leads to elevated wanting, and hence consumption, with this being independent of liking.

We also measured snack-food related memory, as intake recall accuracy has been implicated as a causal pathway by which earlier eating with TV might affect later food intake (Higgs and Woodward, 2009; Mittal et al., 2010; Higgs, 2015). At least four studies – which include two of our own – have found evidence that TV can impair recall accuracy and reduce memory vividness for the TV-paired meal (see Robinson et al., 2013). We found no evidence that recall accuracy was related to variability in lunch intake. However, we did find that higher levels of recall inaccuracy were strongly associated with greater snack food intake. This suggests that previous observations of TVs memory-based effects *may* result indirectly from greater food intake rather than directly from impairing participants’ capacity to encode food-related information. Thus, anything that acts to increase food intake should also have detrimental consequence for intake recall, leading to an underestimation of prior food consumption. This may be one reason that individuals who tend to consume more food, may be those at greatest risk of under-reporting their actual food intake.

## Conclusion

We found that women and men responded differently to the effects of TV on both immediate and delayed food intake. For processed-food intake history, habitually consuming processed foods was associated with greater intake of snack foods largely irrespective of other variables. Finally, we found food recall accuracy was proportional to food intake, with greater intake leading to greater inaccuracy. This suggests one reason why TV, via increased intake, could appear to affect food-related memory.

## Ethics Statement

The study was carried out in accordance with the recommendations of the Macquarie University Human Research Ethics Committee, with written informed consent from all subjects. All subjects gave written informed consent in accordance with the Declaration of Helsinki. The protocol was approved by the Macquarie University Human Research Ethics Committee.

## Author Contributions

HF, RS, MO, MM, and MY conceived and designed the study. HF and RS conducted the study and primary analyses. HF and RS wrote the initial draft manuscript, with MO, MM, and MY commenting and revising this document.

## Conflict of Interest Statement

The authors declare that the research was conducted in the absence of any commercial or financial relationships that could be construed as a potential conflict of interest.
